# Spatial characterization of the effect of age and sex on macular layer thicknesses and foveal pit morphology

**DOI:** 10.1371/journal.pone.0278925

**Published:** 2022-12-15

**Authors:** David Romero-Bascones, Unai Ayala, Ane Alberdi, Asier Erramuzpe, Marta Galdós, Juan Carlos Gómez-Esteban, Ane Murueta-Goyena, Sara Teijeira, Iñigo Gabilondo, Maitane Barrenechea

**Affiliations:** 1 Biomedical Engineering Department, Faculty of Engineering (MU-ENG), Mondragon Unibertsitatea, Mondragón, Spain; 2 Ophthalmology Department, Cruces University Hospital, Barakaldo, Spain; 3 Neurodegenerative Disease Research Group, Biocruces Bizkaia Health Research Institute, Barakaldo, Spain; 4 Department of Neurosciences, Faculty of Medicine and Nursery, University of the Basque Country (UPV/EHU), Leioa, Spain; 5 Ikerbasque, The Basque Foundation for Science, Bilbao, Spain; University of California San Diego, UNITED STATES

## Abstract

Characterizing the effect of age and sex on macular retinal layer thicknesses and foveal pit morphology is crucial to differentiating between natural and disease-related changes. We applied advanced image analysis techniques to optical coherence tomography (OCT) to: 1) enhance the spatial description of age and sex effects, and 2) create a detailed open database of normative retinal layer thickness maps and foveal pit shapes. The maculae of 444 healthy subjects (age range 21–88) were imaged with OCT. Using computational spatial data analysis, thickness maps were obtained for retinal layers and averaged into 400 (20 x 20) sectors. Additionally, the geometry of the foveal pit was radially analyzed by computing the central foveal thickness, rim height, rim radius, and mean slope. The effect of age and sex on these parameters was analyzed with multiple regression mixed-effects models. We observed that the overall age-related decrease of the total retinal thickness (TRT) (-1.1% per 10 years) was mainly driven by the ganglion cell-inner plexiform layer (GCIPL) (-2.4% per 10 years). Both TRT and GCIPL thinning patterns were homogeneous across the macula when using percentual measurements. Although the male retina was 4.1 μm thicker on average, the greatest differences were mainly present for the inner retinal layers in the inner macular ring (up to 4% higher TRT than in the central macula). There was an age-related decrease in the rim height (1.0% per 10 years) and males had a higher rim height, shorter rim radius, and steeper mean slope. Importantly, the radial analysis revealed that these changes are present and relatively uniform across angular directions. These findings demonstrate the capacity of advanced analysis of OCT images to enhance the description of the macula. This, together with the created dataset, could aid the development of more accurate diagnosis models for macular pathologies.

## Introduction

The macula is an approximately 5.5 mm diameter oval pigmented structure of the retina located near its center that is responsible for central high-resolution vision and color perception [1]. Compared to the peripheral retina, the cellular arrangement of the macular retina is unique: it has a high density of photoreceptors and a characteristic centripetal displacement of inner cellular layers that form a concave depression in its center called the fovea, upon which the visual axis is fixed. The macula has a crucial significance in human vision, indeed, pathologies affecting its integrity (e.g., age-related macular degeneration) can lead to severe visual impairment [2].

Optical coherence tomography (OCT)—a non-invasive imaging technique capable of obtaining micrometer resolution images of the retinal structure—has greatly facilitated the investigation of disease-related changes in the structural features of the macula [3]. From OCT images, computational image processing techniques have revealed alterations in macular layer thicknesses [4–6] as well as foveal pit morphology [7, 8] due to ophthalmological and neurological conditions.

**Fig 1:**
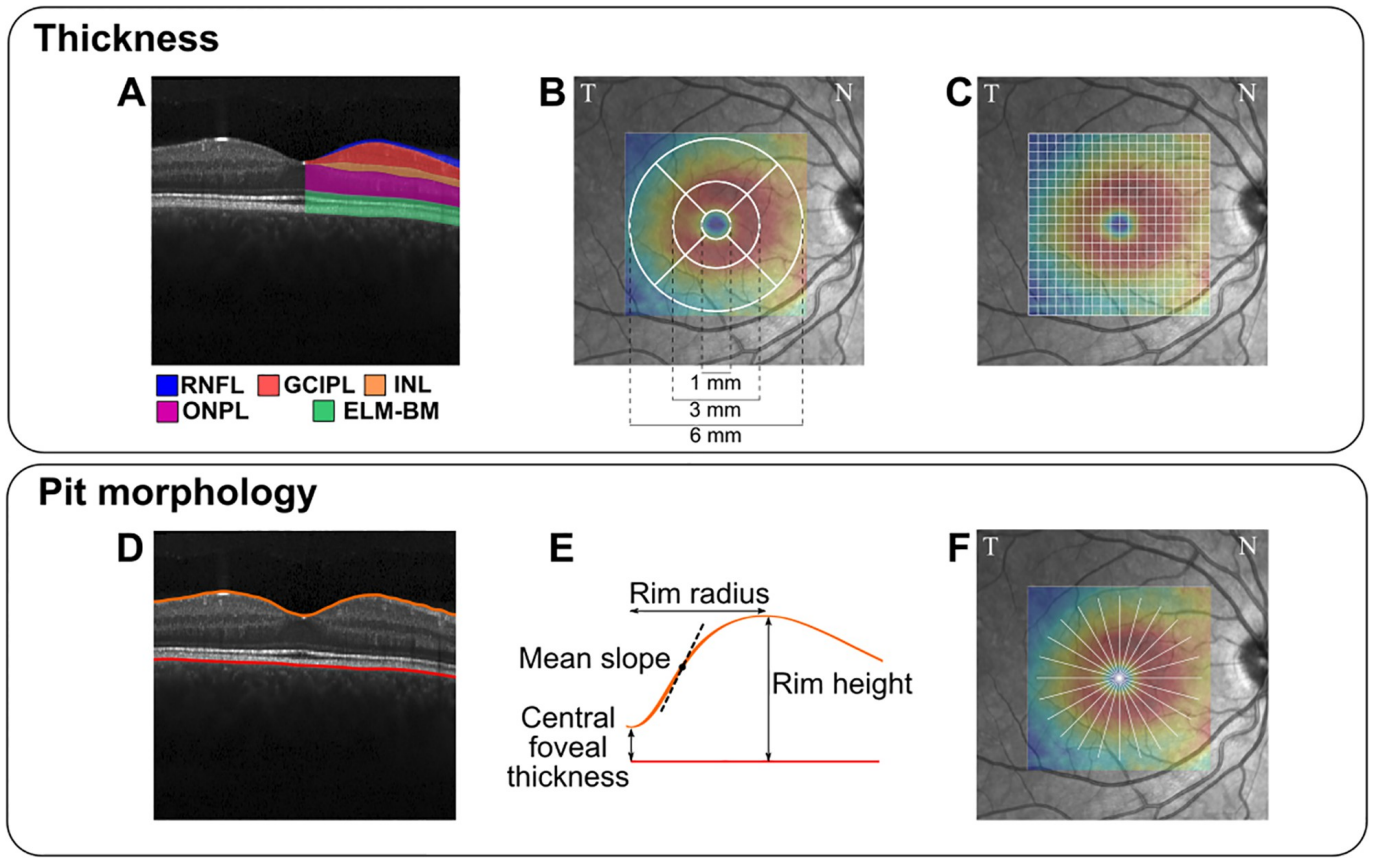
Summary of parameters extracted from macular OCT images. (A) Retinal layers: retinal nerve fiber layer (RNFL), ganglion cell–inner plexiform layer (GCIPL), inner nuclear layer (INL), outer nuclear and plexiform layer (ONPL), and external limiting membrane–Bruch’s membrane complex (ELM-BM). (B) ETDRS sectorization. (C) 20 x 20 square grid sectorization. (D) Segmentation of top and bottom boundaries. (E) Studied foveal pit geometrical descriptors. (F) Foveal pit radial analysis.

Importantly, studies on healthy cohorts have shown that demographical factors such as age, sex, and ethnicity influence the structural parameters of the macula obtained from OCT images. For instance, age-related thinning of the inner retinal layers has been reported in a review [9]. Sex-differences in both thickness and foveal pit geometry have also been detected [10–12]. These findings evidence the importance of accurately characterizing the variation of the retinal structure in healthy populations so that robust conclusions can be reached from clinical studies. In this regard, current knowledge on the matter is limited by several aspects. First, most studies analyzing macular thickness have relied on the standard Early Treatment Diabetic Retinopathy Study (ETDRS) sectorization (Fig 1B) [13–22]. This limits the description of the macular region to the average measurements of nine sectors within a 6-mm circle centered on the fovea. This choice might undermine the ability to capture more detailed spatial variation patterns and recent studies have begun to use smaller than ETDRS sectors. For instance, in [23] authors used 64 sectors (8 x 8 square grid) and found specific clustered spatial patterns of age changes. Similarly, the same 8 x 8 grid was used to establish a normative database of macular thickness in [24]. Additionally, a radial grid with 61 sectors has also been used to examine age changes in retinal thickness [25].

On the other hand, the limited published data available would suggest that the effect of both sex and age on the foveal pit geometry has only been partially studied to date. In the case of sex, after early work on the topic [10, 26], the work of Scheibe et al. was the first relatively large study reporting clear sex differences in foveal pit morphology [11]. More recently, sex differences in foveal curvature were also found in a large study using the UK-Biobank dataset [27]. As for age, this factor has been less explored and large studies have focused mainly on the foveal slope [27–29]. Importantly, only a few works have investigated the foveal shape across multiple angular directions [11, 30].

In light of this gap, we studied both retinal thickness and foveal pit morphology in a large sample of 444 healthy subjects. Following a multiscale approach, we evaluated age-related changes and sex differences on retinal thickness using both the regular ETDRS and a 20 x 20 square grid sectorizations. With the latter, we aimed to provide a more detailed normative database by using smaller sectors than those existing in the literature. Similarly, we assessed the effect of such demographic factors on the central thickness, rim height, radius, and slope of the foveal pit measured for the whole macula as well as for 24 angular directions individually. With this characterization we extend existing knowledge by examining changes in various aspects of the fovea with high spatial detail.

## Materials and methods

### Participants

A total of 444 healthy subjects (855 eyes) were included in the study. Subjects were recruited at the Ophthalmology and Neurology Departments of the Cruces University Hospital (Barakaldo, Spain). The mean age of participants was 54.9 ± 12.7 years (ranging from 21 to 88) and 63% were females (see Table 1 for a summary and S1 Fig for the detailed age distribution). All subjects were Caucasian. Before inclusion in the study, all participants underwent a screening process that consisted of an ophthalmological examination and a comprehensive questionnaire on neurological, systemic, and eye-related diseases. We excluded subjects with a history of heavy smoking (>20 cigarettes/day), heavy alcohol use (>4 drinks/day for men or >3 drinks/day for women), diagnosis of any type or grade of diabetes, uncontrolled or resistant elevated blood pressure, obesity (body mass index > 30), history of consumption of drugs or medications known to induce retinal toxicity, chronic inflammatory systemic diseases, history of traumatic brain injury, or neurological diseases. Additionally, we excluded subjects with spherical equivalent refractive error > 4.00 diopters or < -4.00 diopters, >3.00 diopters of astigmatism, or any other ocular condition potentially affecting OCT measures, as detailed in the OSCAR-IB consensus criteria [31]. In cases where only one of the eyes of a participant was excluded, the other eye was included. Following the tenets of the Declaration of Helsinki, all participants gave written informed consent prior to their participation. The study was approved by the Institutional Ethics Committee of OSI Ezkerraldea-Enkarterri-Cruces (Barakaldo, Spain).

**Table 1:** Subject demographic data.

Group	Subjects	Eyes	Age	Subjects by age group (years old)			
				< 40	40–60	60–80	>80
All	444	855	54.9 ± 12.7 [21, 88]	51	212	161	8
Female	281	543	54.3 ± 12.6 [22, 88]	34	135	101	3
Male	163	312	56.0 ± 12.8 [21, 87]	17	77	60	5

Age in format mean ± standard deviation [range]

### Image acquisition and processing

The eyes of all subjects were imaged using a Spectralis spectral domain OCT scanner (Heidelberg Engineering, Heidelberg, Germany) following a macular raster acquisition protocol with a 20º field of view, 25 horizontal B-scans, and 512 A-scans per B-scan. For each final B-scan, a total of 49 B-scans were averaged. No pupil dilation was employed and default keratometry values were used. Additionally, both eyes of a subset of 12 subjects were imaged for a second time, covering the same macular area but following a higher resolution protocol with 97 B-Scans and 1024 A-scans. All other parameters of the second scans were the same. This dataset was used to investigate the impact of different acquisition protocols in a sensitivity analysis. Ocular magnification was corrected by the built-in Spectralis software [32]. All images were segmented with Heidelberg Eye Explorer 1.9.10.0 software. Segmentations were reviewed by three specialists (A.M., S.T. and I.G.) and evident errors within the 3 mm radius macular region were manually corrected. The images were loaded into MATLAB 2020b (The Mathworks Inc., Natick, MA, United States) and subsequently analyzed using the open-source RETIMAT Toolbox (https://github.com/drombas/retimat). All scans were aligned by automatically locating the foveal center as the minimum value of a smoothed total retinal thickness (TRT) map [33]. Left eyes were flipped to match right eyes. From the segmentation data, two macular features were studied: retinal layer thicknesses and foveal pit morphology.

The retinal layers depicted in Fig 1A were studied: TRT, retinal nerve fiber layer (RNFL), ganglion cell–inner plexiform layer (GCIPL), inner nuclear layer (INL), outer nuclear and plexiform layer (ONPL), and external limiting membrane–Bruch’s membrane complex (ELM-BM). Point thickness values were interpolated to a 300 x 300 regular grid, with a spacing of 0.1 mm. Then, these values were averaged following three fovea-centered sectorizations: whole macular region (i.e., the 3 mm radius circular region), ETDRS (Fig 1B), and a 6 x 6 mm sectorization with 20 x 20 square sectors (Fig 1C). In this latter sectorization, regions outside of the 3 mm fovea-centered circle were excluded. Similarly, the thicknesses of the RNFL, GCIPL, and INL layers were not analyzed in the centermost sectors (i.e., the central 1.2 x 1.2 mm region). This is because the thicknesses of the inner layers in the central foveal region are almost zero and any results including these sectors could be highly biased by segmentation errors.

Regarding foveal pit morphology, TRT point values were interpolated to a radial grid with 24 angular directions, 2 mm radius, and 0.1 mm spacing. First, the central foveal thickness (CFT) was estimated as the TRT at the foveal center. LOESS smoothing (span = 50%) was then separately applied to the TRT profile for each radial direction to reduce the ripple before computing the rim height as the point of maximum TRT. Based on the rim height, two additional parameters were derived: rim radius i.e., the lateral distance from the foveal center to the rim, and mean slope i.e., the mean first derivative of the unsmoothed TRT between the foveal center and the rim (Fig 1D). Except for the CFT, which does not vary radially, all parameters were computed for both the whole macula (i.e., averaging all 24 directions) and for every single direction. S2 Fig illustrates this process.

### Data analysis

The effects of sex and age on thickness maps and foveal pit parameters were studied in both absolute and percentual units by means of multivariate regression. The models included fixed terms for age and sex (with females as the reference). To adjust for differences in ocular shape, a fixed term for the scan focus variable was also included. This variable is estimated by the Spectralis scanner while focusing the image and accounts for the refraction error of each eye, which is known to influence retinal measurements. Importantly, a mixed-effects model variant with a random intercept (*γ*_*subject*_) was used to account for the inter-eye correlation. As a first model selection step, two models with linear (Eq 1) and quadratic (Eq 2) age effects were fitted and the one with the minimum Akaike information criteria was chosen.


$$y=\beta_{0}+\beta_{sex}isMale+\beta_{age}age+\beta_{sf}scanFocus+\gamma_{subject}$$ (1)

$$y=\beta_{0}+\beta_{sex}isMale+\beta_{age}age+\beta_{age2}age^{2}+\beta_{sf}scanFocus+\gamma_{subject}$$ (2)

To equivalently report results from linear and quadratic models, a single combined age effect coefficient was estimated as the mean yearly change between the age of 40 and 80. For each coefficient, a 95% confidence interval (CI) and a p-value were calculated. When the selected model was quadratic, a single p-value was computed for the combined linear + quadratic effect of age by using an F-test to compare a reference model without any age term with the quadratic model. Age coefficients were transformed into % values by dividing the estimates by the average parameter value in the youngest group (age < = 40, including n = 51 from the 444 subjects). Likewise, sex coefficients were divided by the estimate for females in the youngest group (age < = 40, n = 37) to obtain % values. The significance level was set to 0.05. Results for the whole macular region were adjusted for multiple comparisons based on the Holm-Bonferroni correction [34]. For both ETDRS and the high-resolution sectorizations, a correction based on the false discovery rate [35] was employed due to the high number of sectors and the potential statistical dependence between tests. The overall fit of the model was assessed by the marginal R-squared, which measures the variance explained solely by the fixed terms. The age coefficient was reported as changes per 10 years as annual changes were found to be very small.

Using the subset of subjects imaged twice, we compared the measurements obtained by the standard protocol (25 B-scans, 512 A-scans) with the high-resolution protocol (97 B-scans, 1024 A-scans). For every parameter, a mixed-effects model linear regression was fitted with a fixed term for the bias introduced by using the standard protocol (*β*_*bias*_) and a random intercept (*γ*_*subject*_) to account for inter-eye correlation:

$$y=\beta_{0}+\beta_{bias}isStandard+\gamma_{subject}$$ (3)

A p-value as well as a 95% confidence interval (CI) were computed for the bias term. Finally, the estimated bias was divided by the intercept to obtain a percentual bias.

## Results

### Effects of age and sex on macular thickness

**Fig 2:**
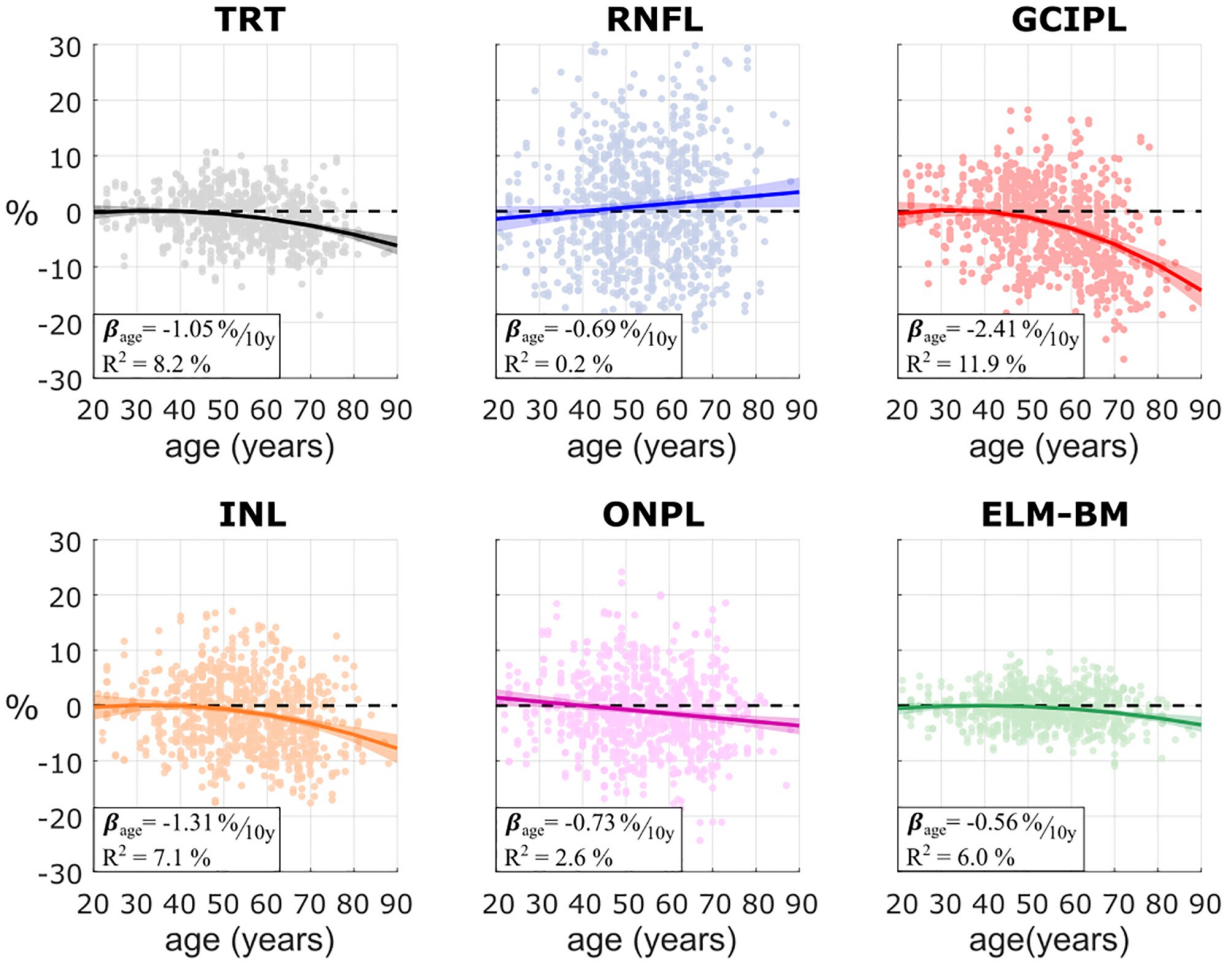
Percentual change in retinal thicknesses as a function of age for the 3 mm radius circular macular region. Individual absolute thickness values were transformed into percentages as the relative difference with respect to the average thickness in the youngest group (age < 40). Layers under study: total retinal thickness (TRT), retinal nerve fiber layer (RNFL), ganglion cell–inner plexiform layer (GCIPL), inner nuclear layer (INL), outer nuclear and plexiform layer (ONPL), and external limiting membrane–Bruch’s membrane complex (ELM-BM).

Fig 2 shows the mean thickness as a function of age on each layer for the whole macular region. Table 2 reports the regression coefficient estimates along with the corresponding p-values and the R^2^ of the model. All layers were found to become significantly thinner with age except for the RNFL (with a non-significant thickening and a high dispersion) and the ELM-BM. The thinning effect was stronger for the TRT, GCIPL, and INL. From these, the GCIPL presented the highest percentual loss (-2.4 [-2.9, -1.9] % every 10 years) and was responsible for most of the reduction in the TRT (-1.1 [-1.3, -0.8] % every 10 years).

**Table 2:** Regression results of mean macular layer thickness.

Layer	Age dependence	Age			Sex			R^2^ (%)
		β [95% CI]		p	β [95% CI]		p	
		μm / 10 years	% / 10 years		μm (male)	% (male)		
TRT	Quadratic	-3.25 [-4.09, -2.39]	-1.05 [-1.32, -0.77]	9·10^−13^*	4.14 [1.78, 6.5]	1.34 [0.58, 2.11]	5·10^−4^*	8.2
RNFL	Linear	0.22 [-0.04, 0.48]	0.69 [-0.14, 1.52]	0.1	-0.03 [-0.71, 0.65]	-0.1 [-2.22, 2.03]	0.9	0.2
GCIPL	Quadratic	-1.77 [-2.13, -1.4]	-2.41 [-2.90, -1.90]	3·10^−22^*	0.57 [-0.41, 1.56]	0.78 [-0.56, 2.13]	0.3	11.9
INL	Quadratic	-0.45 [-0.6, -0.3]	-1.31 [-1.74, -0.88]	9·10^−9^*	0.87 [0.46, 1.28]	2.57 [1.36, 3.77]	3·10^−5^*	7.1
ONPL	Linear	-0.66 [-1.12, -0.19]	-0.73 [-1.24, -0.21]	0.006*	1.8 [0.59, 3.02]	2.01 [0.66, 3.36]	0.004*	2.6
ELM-BM	Quadratic	-0.45 [-0.63, -0.26]	-0.56 [-0.78, -0.33]	2·10^−4^*	0.93 [0.48, 1.39]	1.17 [0.6, 1.74]	7·10^−5^*	6.0

For each layer, the regression coefficients (*β*_*age*_ and *β*_*sex*_) are presented in both absolute and percentual units. The statistical evidence is evaluated by a 95% confidence interval (CI) and uncorrected p-values. Statistically significant p-values after Holm-Bonferroni correction (number of tests: 12, α = 0.05) are marked with an asterisk (*). The effect values of the sex variable are represented as the difference of male minus female. Abbreviations: TRT: total retinal thickness; RNFL: retinal nerve fiber layer; GCIPL: ganglion cell–inner plexiform layer; INL: inner nuclear layer; ONPL: outer nuclear and plexiform layer; ELM-BM: external limiting membrane–Bruch’s membrane complex; R^2^: marginal R-squared.

On the other hand, male retinas were found to be 4.1 [1.8, 6.5] μm thicker on average (Table 2). This was a combined effect of differences in all individual layers, although the INL, ONPL, and ELM-BM were the only layers that showed statistically significant differences between males and females.

**Fig 3:**
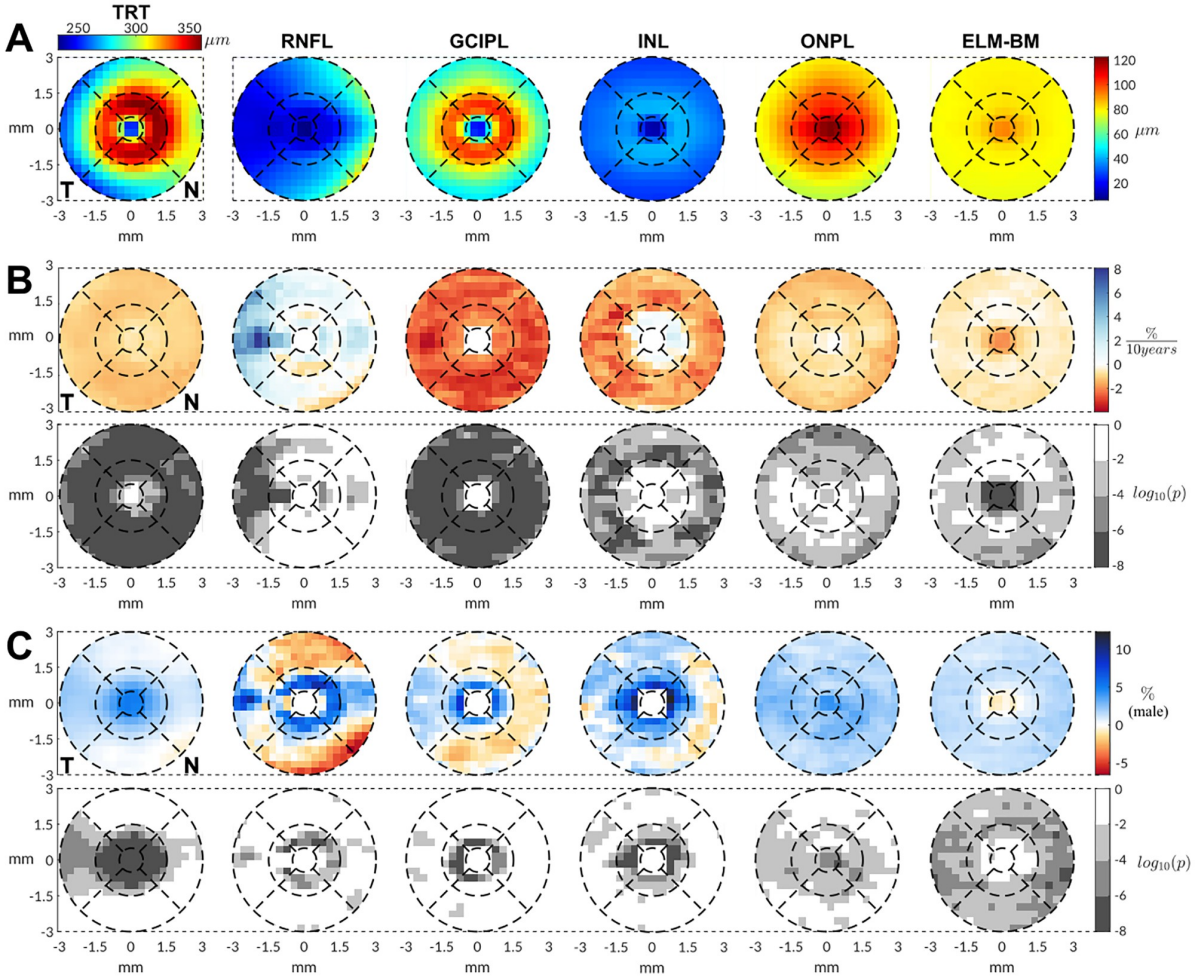
Thickness analysis results for the 20 x 20 regular grid sectorization. (A) Population mean thicknesses. (B) Age-related changes of retinal layer thicknesses measured as % of change per 10 years (top) and corresponding p-values (bottom). (C) Sex differences in percentual units for males (top) and associated p-values (bottom). Percentual values were calculated after transforming absolute thickness values into the relative difference with respect to the average thickness of participants younger than 40 years old. P-values are reported in logarithmic scale after false discovery rate correction. Layers under study: total retinal thickness (TRT), retinal nerve fiber layer (RNFL), ganglion cell–inner plexiform layer (GCIPL), inner nuclear layer (INL), outer nuclear and plexiform (ONPL), and external limiting membrane–Bruch’s membrane complex (ELM-BM).

When the spatial distribution of thickness changes in relation to age and sex on the 20 x 20 grid was analyzed (Fig 3), we observed that the age-related thinning of the TRT, GCIPL and ONPL was relatively homogeneous across the macular area. For the INL, however, the age-related reduction in thickness was more localized in the outer ring of the macula (perifoveal region). In the case of the RNFL maps, there was a mild thickening which was especially prominent in the temporal sector. Finally, the changes in the ELM-BM were minor and not significant except for the foveal central region where a slight-moderate thinning was observed. The regions in which a quadratic model was selected are depicted in S3 Fig.

With regards to sex, differences in the TRT were prominent in the central region (radius<1.5 mm), with males having up to a 4% thicker retina. As for the individual layers, the sex differences in the RNFL, GCIPL, and INL layers were more obvious in the inner ring (0.5 mm ≤ radius ≤ 1.5 mm), where thickness values in males were also significantly higher than in females. At larger radii, these differences diminished and even reversed. Sex differences in the ONPL and ELM-BM layers were less pronounced and more homogeneous in percentage terms. However, they remained significant (males > females), predominantly for the outer ring (perifovea) in the ELM-BM and for the inner ring (parafovea) in the ONPL. For completeness, full regression coefficients including linear, quadratic and scan focus terms as well as correspondent results for ETDRS sectorization are reported as S1 Appendix.

### Effects of age and sex on foveal pit morphology

Both sex and age influenced the foveal pit morphology (Table 3 and S4 Fig). The rim height showed a statistically significant age-related decrease of 0.97 [0.71, 1.22] % every 10 years. In contrast, the CFT, rim radius, and especially the mean slope, presented important inter-subject variability and no clear age effect was detected. Sex-related differences were evident in all the parameters. Males had a larger CFT (+8.0 [4.2, 11.8] μm), a higher rim height (+8.6 [5.9, 11.3] μm), a shorter rim radius (-59.4 [-78.3, -40.4] μm), and a steeper mean slope (+0.4 [0.2, 0.6] º). Percentage differences were greatest for the mean slope (6.2 [2.8, 9.6] %).

**Table 3:** Regression results for foveal pit morphology.

Parameter	Age dependence	Age			Sex			R^2^ (%)
		β [95% CI]		p	β [95% CI]		p	
		X / 10 years	% / 10 years		X (male)	% (male)		
CFT (μm)	Quadratic	-1.44 [-2.93, 0.03]	-0.62 [-1.25, 0.01]	0.08	7.99 [4.22, 11.77]	3.47 [1.83, 5.11]	3·10^−5^*	4.8
Rim height (μm)	Quadratic	-3.42 [-4.34, -2.51]	-0.97 [-1.22, -0.71]	10^−11^*	8.63 [5.94, 11.33]	2.46 [1.69, 3.23]	5·10^−10^*	11.9
Rim radius (μm)	Linear	-7.66 [-14.99, -0.33]	-0.69 [-1.36, -0.03]	0.04	-59.4 [-78.34, -40.46]	-5.24 [-6.91, -3.57]	1·10^−9^*	8.4
Mean slope (º)	Linear	-0.06 [-0.15, 0.02]	-0.99 [-2.27, 0.29]	0.15	0.39 [0.18, 0.6]	6.23 [2.84, 9.63]	3·10^−4^*	4.1

For each parameter, the regression coefficients (*β*_*age*_ and *β*_*sex*_) are presented in both absolute and percentual units. The statistical evidence is evaluated by a 95% confidence interval (CI) and uncorrected p-values. Statistically significant p-values after Holm-Bonferroni correction (number of tests = 8, α = 0.05) are marked with an asterisk (*). Abbreviations: CFT: central foveal thickness; R^2:^ marginal R-squared.

**Fig 4:**
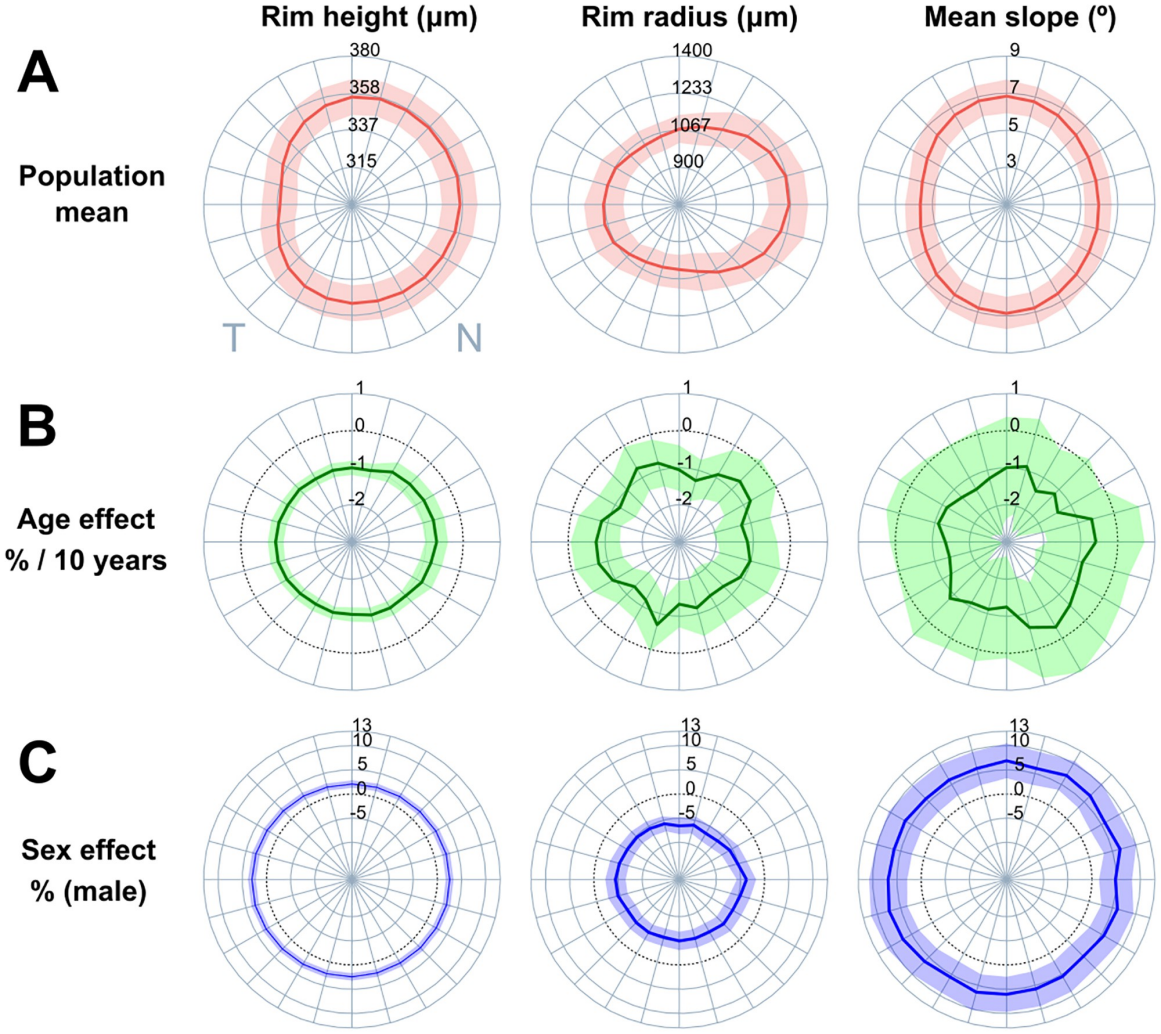
Radial analysis of rim height, rim radius and mean slope. (A) Population mean (central colored line indicates the mean, shaded region depicts the 2.5 and 97.5 percentiles). Percentual effect of age (B) and sex (C) are shown as the normalized regression coefficients (β_age_ and β_sex_) for each of the 24 angular directions. The shaded region illustrates the 95% confidence interval while the dashed black circle locates the origin (coefficients equal to zero).

The observed age and sex differences were also found when the foveal morphology was studied radially (Fig 4). More specifically, the estimated reduction in rim height was present in all directions and ranged from 0.6% to 0.8% per 10 years. The rim radius was found to decrease in all directions, although the effect was stronger in the inferior and nasal sectors, with a 1% decrease per 10 years. Conversely, sex-related differences were clearly present in every angular direction. The differences were relatively uniformly distributed except for the rim radius, which showed slightly larger differences for the superior and inferior sectors.

### Sensitivity analysis

The estimated bias introduced in each average thickness and foveal parameter due to the resolution of the acquisition protocol is shown in Table 4. Corresponding results for ETDRS and 20 x 20 sectorizations can be found in S1 Appendix and S5 Fig, respectively. The results highlight that the bias is small for average macular thickness (< 1%) but is not negligible for central thickness (3.02%) and foveal slope (-6.57%). Importantly, when using smaller sectors this bias is below 5% overall with the centermost and outer regions being the most affected.

**Table 4:** Sensitivity analysis results for average macular and foveal parameters.

Family	Layer/Parameter	Absolute bias	Relative bias (%)	P-value
Thickness (μm)	TRT	0.1 [-0.93, 1.14]	0.03 [-0.31, 0.37]	0.84
	RNFL	0.11 [-0.37, 0.59]	0.36 [-1.19, 1.9]	0.64
	GCIPL	-0.4 [-0.88, 0.08]	-0.57 [-1.26, 0.11]	0.099
	INL	0.02 [-0.27, 0.32]	0.07 [-0.83, 0.97]	0.87
	ONPL	-0.18 [-0.59, 0.24]	-0.19 [-0.64, 0.26]	0.4
	ELM-BM	0.55 [0.11, 0.98]	0.69 [0.14, 1.24]	0.015*
Foveal pit morphology	Central foveal Thickness (μm)	6.72 [4.88, 8.57]	3.02 [2.19, 3.84]	3·10^−9^*
	Rim height (μm)	-0.79 [-2.34, 0.75]	-0.23 [-0.67, 0.21]	0.31
	Rim radius (mm)	-0.01 [-0.04, 0.01]	-1.26 [-3.23, 0.7]	0.2
	Mean slope (º)	-0.45 [-0.65, -0.25]	-6.57 [-9.51, -3.64]	5·10^−5^*

The statistical evidence is evaluated by 95% confidence interval and p-values. Statistically significant p-values are marked with an asterisk (*). Abbreviations: TRT: total retinal thickness; RNFL: retinal nerve fiber layer; GCIPL: ganglion cell–inner plexiform layer; INL: inner nuclear layer; ONPL: outer nuclear and plexiform layer; ELM-BM: external limiting membrane–Bruch’s membrane complex.

## Discussion

In this study, we evaluated the influence of age and sex on the structure of the retina by testing their relationship with finely sectorized thickness maps of macular layers and foveal pit morphology metrics extracted from OCT images of 444 healthy subjects (855 eyes). We first observed that in relation to age there is a homogeneous TRT thinning of 1.1% per 10 years, driven mainly by the GCIPL (-2.4%) followed by the INL (-1.3%) and ONPL (-0.7%). The rim height decreased significantly with age at a rate of 1.0% every 10 years. Second, we found that on average male retinas were 4.1 μm thicker, with more prominent differences in the central region (radius<1.5 mm). Males had a larger central CFT, higher rim height, shorter rim radius and a steeper mean slope.

The observed age-related TRT decline portrays the retina as an evolving structure, a finding that is well supported in the literature [9, 15, 18–21]. In particular, one review of the literature described the age-related thinning pattern as spatially-dependent, with an unchanged or thickened central retina, and a maximum thickness loss in the parafoveal region [9]. In the present study, we focused on the percentage loss of TRT and found a relatively uniform thinning effect for eccentricities larger than 0.5 mm. This suggests that, except for the central macula, differences in the absolute thinning rate can be explained by differences in baseline TRT thickness.

Of greater interest than the TRT analysis, however, is the determination of the individual layers (with specific cellular architectures and spatial distributions) that drive the thinning effect. In this regard, histological studies have observed an age-related decrease in the number of fibers that comprise the optic nerve and the RNFL [36, 37]. In contrast, we found that the RNFL remained unchanged or even became thicker in some temporal regions. This discrepancy is also present in the literature, with different studies reporting thinning [22, 38], no effect [18, 19], or thickening [20, 39]. These mixed results might be explained by the thinness of the RNFL in relation to the axial resolution, which makes thickness measurements more prone to segmentation errors. Therefore, using peripapillary instead of macular OCT seems more appropriate to assess the RNFL.

As for the GCIPL, the thinning we observed is supported by histological evidence that points to an age-related decrease in ganglion cell density [37]. Similarly, the majority of previous studies reported a thinning of both the ganglion cell layer (GCL) [22, 38, 40] and the inner plexiform layer (IPL) [17, 20, 21, 25]. We found the GCIPL to be the layer most affected by age, which may indicate that it is particularly sensitive to aging. Additionally, we measured a consistent GCIPL percentual loss across the macula, except for the central region. These results are in line with previous work [41, 42] and suggest that regional differences in absolute thickness loss—such as the accentuated parafoveal thinning [25]—reflect differences in absolute GCL thickness and not a spatially-dependent predisposition to an age-related decline. As for the INL, a thinning effect has been previously reported [18, 20, 21, 25]. In a similar vein, our results describe the INL as the layer with the second most important age decline and a prevalent thinning of the outer regions. Finally, and in contrast to the thickening of the outer retinal layers described previously [9], we measured a thinning of both the ONPL and the ELM-BM, a finding more in line with recent studies [21, 25, 39]. Interestingly, we found two different thinning patterns: a prevalent and uniform ONPL thinning of outer regions, and a highly localized central region ELM-BM thinning. The different cellular configurations of these layers might explain these characteristic patterns.

Although the repeatability of Spectralis has been reported to be very good [43], it is important to note that the observed yearly changes are, for the most part, small when compared to the coefficient of variation. For instance, the coefficient of variation of Spectralis TRT measurements (with eye-tracking mode) is up to 0.86% [44], which would correspond to a decade change solely due to age. This underscores two points: 1) natural age changes would have a relatively small impact on longitudinal studies with a regular follow up (e.g., < 5 years) and 2), despite its high repeatability, OCT requires large sample sizes and groupwise statistical analysis to detect small changes in the retina.

Regarding sex differences, a thicker retina in males has been previously reported in both adults [13–15, 18] and children [45]. In all studies, differences were higher for the inner macular ring and diminished for the outer ring, a pattern also observed in our analysis. In addition, our analysis revealed spatially localized differences in all layers, which suggests that sex plays a role in the entire retina. As further evidence of this, layer-specific differences have been described previously. For instance, some studies measured a thicker RNFL in males [17, 18, 21, 24], while others observed it to be thicker in females [19, 22, 38]. In addition, a greater thickness in males has been found for the GCL [16, 18, 21, 24], inner plexiform layer [17, 18, 21] and INL [19]. As for the outer retinal layers, sex differences have also been reported [17, 19].

A possible explanation for this thicker retina in males could be a systematic macroscopic difference. In fact, a clear finding from magnetic resonance imaging studies is that males have a larger ocular globe [46] and, therefore, one could hypothesize that larger eyes have a thicker retina. Contrary to this, a negative relationship between axial length and retinal thickness has been observed [47], although this might be confounded by the ocular effect of ocular biometry on lateral image scaling [48, 49] (i.e., the same field of view corresponds to a larger region in larger eyes).

Similarly, a sex bias in lateral image scale estimation could explain why sex differences in inner layer thicknesses are localized in the inner ring. Although lateral scaling is usually adjusted for axial length differences [10, 21], scanners typically assume a nominal corneal curvature value for both males and females (e.g. Spectralis uses a 7.7 mm [32]). This assumption might lead to an overestimation of the lateral scaling in females, as corneal curvature is both positively related with lateral scaling and smaller in females [50]. This, in turn, could displace the maxima of the TRT, GCIPL and INL thickness profiles in females to larger eccentricities, thereby resulting in the observed pattern.

With respect to the effect of age on the foveal pit, neither previous studies [26, 30] nor our data showed a clear effect on the CFT (S1 Table). This is likely to be related to the absence of inner retinal layers in the central region, which are more prone to becoming thinner [9]. Regarding the rim height, although smaller previous studies did not find a statistically significant effect [26, 30] we observed a clear thinning of the rim, which is in line with the known TRT loss. In addition, we also observed a decrease in the rim radius, which might be a consequence of a flattening of the rim due to the rim height decrease. This, however, did not remain statistically significant after correction. The high inter-subject variability of the mean slope resulted in high uncertainty in the estimates which is likely responsible for the mixed results in the literature [12, 28, 29].

Although existing studies describing sex differences in the foveal pit used different parameter definitions and mathematical models, one finding is consistent between our results and most of the previous work: a broader and shallower pit in females [10–12, 27, 51] (S2 Table). As with thickness differences, the lateral scale estimation bias introduced by ocular magnification might lead to an overestimation of the lateral scaling in females and the observed differences in slope and radius. As for height measurements, our study confirms previous findings of greater CFT and rim height in males [11]. Considering that these foveal pit metrics are effectively thickness measurements, it is likely that these differences are simply a consequence of a higher TRT in males.

In addition to the overall description, the foveal pit is known to be a radially asymmetric structure—it is broader in the horizontal plane compared to the vertical directions [11]. We studied 24 angular directions individually and found that percentual age and sex effects are relatively uniform across all directions. This can indicate that, despite its structural asymmetry, the fovea is a homogeneously evolving structure.

This study is not without limitations. First, using 25 B-scans under-samples the central region, which introduces a systematic overestimation and underestimation of central thicknesses and foveal slope measurements, respectively. While this is important to consider, the sensitivity analysis also revealed that the bias in most of the estimations is relatively low (<5%). We followed our standard 25-Bscan protocol as it is used in regular practice and requires a shorter scanning time, which is crucial when imaging subjects with neurodegenerative diseases. Second, we employed raster scans and interpolation to analyze the fovea radially instead of using a radial acquisition pattern. Although this may hinder an accurate reconstruction of the TRT profile in the vertical direction, we selected it because radial patterns–with an irregular sampling density–could potentially introduce a bigger bias when measuring thicknesses far from the central region or when correcting fixation errors.

We did not correct for display distortion, as neither biometric nor scanner optical information was available [49]. The errors due to such distortion are minimal for thickness measurements and small fields of view like the one used in this study [48] but have an impact on slope metrics [52]. We addressed the latter by computing the slope after flattening the retina (i.e., using the TRT). Although this procedure does not measure the actual slope seen in OCT images, it is the most common approach as it helps minimize the effect of both retinal curvature and display distortion.

Axial length is also known to influence retinal measurements. To address this, we adjusted all regression models using the scan focus, a parameter exported by the scanner that is measured when focusing the image and accounts for the refractive error of each eye [32]. Given that refractive error is closely related to axial length (R^2^ > 0.72) [53], we considered the scan focus parameter as a reasonable proxy for axial length. Additionally, we relied on the lateral image scale estimation performed by the Spectralis scanner to correct the ocular magnification problem. This procedure, however, might be limited when using default corneal curvature values [32]. Finally, we did not include an interaction term between sex and age as the high inter-subject variability would diminish the statistical power to detect a potentially very small effect.

## Conclusions

Most retinal layers present thinning over time that is more prominent for the GCIPL. Percentual changes in TRT and GCIPL are homogeneous even when analyzed in very small sectors. Overall, males have thicker retinal layers than females, although the differences are more evident in the inner ring. The clearest effect of age on the foveal pit is a decrease in the rim height. Male and female foveae show evident differences, with females having a shallower and broader pit. Sex and age effects are present for all angular directions. Advanced analysis of OCT images such as highly detailed thickness sectorization or radial geometrical analysis of the foveal pit can be used to enhance the description of the macula.

## Supporting information

S1 Fig Subject demographic data in five-year buckets. (TIF)

S2 Fig Foveal pit morphology analysis pipeline. (TIF)

S3 Fig Selected age model for each 20 x 20 grid sector. (A) R-squared of the linear model (Eq 1 in the paper). (B) Improvement on the R-squared when a quadratic term for age is added (β_age2_·age^2^). (C) Model selected for each sector. Layers under study: total retinal thickness (TRT), retinal nerve fiber layer (RNFL), ganglion cell–inner plexiform layer (GCIPL), inner nuclear layer (INL), outer nuclear and plexiform (ONPL), and external limiting membrane–Bruch’s membrane complex (ELM-BM).
(TIF)

S4 Fig Percentual change in foveal pit morphology parameters as a function of age. Each parameter value was obtained after averaging 24 angular directions and transformed into percentages as the relative difference with respect to the average value of the youngest group (age < 40).
(TIF)

S5 Fig Sensitivity analysis results for 20 x 20 grid thickness analysis. Percentual bias between regular and high-resolution acquisition protocols for the whole macula (A), the ETDRS sectors (B) and the 20 x 20 grid (C). The latter includes the distribution of individual sector values (bottom row).
(TIF)

S1 Appendix Results for ETDRS sectorization and full regression coefficients for macular thickness and foveal pit parameters. (DOCX)

S1 Table Comparison of studies analyzing the effect of age on the foveal pit. *p<0.05. Abbreviations: CFT: central foveal thickness; NAF: no association found, estimations not reported. ^†^In the present study the mean slope was studied instead of the maximum slope. Olvera-Barrios et al. measured foveal curvature instead of slope.
(DOCX)

S2 Table Comparison of studies analyzing sex differences in the foveal pit. The results are reported as the difference in the mean values of males minus females. *p<0.05. Abbreviations: CFT: central foveal thickness. ^†^ In the present study the mean slope was studied instead of the maximum slope. Olvera-Barrios et al. measured foveal curvature instead of slope.
(DOCX)

## References

[1] Atchinson DA, Smith G. Basic optical structure of the human eye. In: Heinemann B, editor. Optics of the human eye. Butterworth-Heinemann; 2000. p. 7.

[2] Lim LS, Mitchell P, Seddon JM, Holz FG, Wong TY. Age-related macular degeneration. Lancet. 2012;379: 1728–1738. doi: 10.1016/S0140-6736(12)60282-7 22559899

[3] Huang D, Swanson EA, Lin CP, Schuman JS, Stinson WG, Chang W, et al. Optical coherence tomography. Science. 1991;254: 1178–1181. doi: 10.1126/science.1957169 1957169 PMC4638169

[4] Hammel N, Belghith A, Weinreb RN, Medeiros FA, Mendoza N, Zangwill LM. Comparing the Rates of Retinal Nerve Fiber Layer and Ganglion Cell–Inner Plexiform Layer Loss in Healthy Eyes and in Glaucoma Eyes. Am J Ophthalmol. 2017;178: 38–50. doi: 10.1016/j.ajo.2017.03.008 28315655

[5] Chan VTT, Sun Z, Tang S, Chen LJ, Wong A, Tham CC, et al. Spectral-Domain OCT Measurements in Alzheimer’s Disease: A Systematic Review and Meta-analysis. Ophthalmology. 2019;126: 497–510. doi: 10.1016/j.ophtha.2018.08.009 30114417 PMC6424641

[6] Chrysou A, Jansonius NM, van Laar T. Retinal layers in Parkinson’s disease: A meta-analysis of spectral-domain optical coherence tomography studies. Park Relat Disord. 2019;64: 40–49. doi: 10.1016/j.parkreldis.2019.04.023 31054866

[7] Ding Y, Spund B, Glazman S, Shrier EM, Miri S, Selesnick I, et al. Application of an OCT data-based mathematical model of the foveal pit in Parkinson disease. J Neural Transm. 2014;121: 1367–1376. doi: 10.1007/s00702-014-1214-2 24748549

[8] Akula JD, Arellano IA, Swanson EA, Favazza TL, Bowe TS, Munro RJ, et al. The Fovea in Retinopathy of Prematurity. Invest Ophthalmol Vis Sci. 2020;61: 28. doi: 10.1167/iovs.61.11.28 32936301 PMC7500148

[9] Subhi Y, Forshaw T, Sørensen TL. Macular thickness and volume in the elderly: A systematic review. Ageing Res Rev. 2016;29: 42–49. doi: 10.1016/j.arr.2016.05.013 27262495

[10] Wagner-Schuman M, Dubis AM, Nordgren RN, Lei Y, Odell D, Chiao H, et al. Race- and sex-related differences in retinal thickness and foveal pit morphology. Investig Ophthalmol Vis Sci. 2011;52: 625–634. doi: 10.1167/iovs.10-5886 20861480 PMC3053303

[11] Scheibe P, Zocher MT, Francke M, Rauscher FG. Analysis of foveal characteristics and their asymmetries in the normal population. Exp Eye Res. 2016;148: 1–11. doi: 10.1016/j.exer.2016.05.013 27191610

[12] Zouache MA, Silvestri G, Amoaku WM, Silvestri V, Hubbard WC, Pappas C, et al. Comparison of the Morphology of the Foveal Pit Between African and Caucasian Populations. Transl Vis Sci Technol. 2020;9: 24. doi: 10.1167/tvst.9.5.24 32821496 PMC7401974

[13] Kashani AH, Zimmer-Galler IE, Shah SM, Dustin L, Do D V., Eliott D, et al. Retinal Thickness Analysis by Race, Gender, and Age Using Stratus OCT. Am J Ophthalmol. 2010;149: 496–512. doi: 10.1016/j.ajo.2009.09.025 20042179 PMC2826608

[14] Song WK, Lee SC, Lee ES, Kim CY, Kim SS. Macular thickness variations with sex, age, and axial length in healthy subjects: A spectral domain-optical coherence tomography study. Investig Ophthalmol Vis Sci. 2010;51: 3913–3918. doi: 10.1167/iovs.09-4189 20357206

[15] Patel PJ, Foster PJ, Grossi CM, Keane PA, Ko F, Lotery A, et al. Spectral-Domain Optical Coherence Tomography Imaging in 67 321 Adults: Associations with Macular thickness in the UK Biobank Study. Ophthalmology. 2015;123: 829–840. doi: 10.1016/j.ophtha.2015.11.009 26746598

[16] Wang J, Gao X, Huang W, Wang W, Chen S, Du S, et al. Swept-source optical coherence tomography imaging of macular retinal and choroidal structures in healthy eyes. BMC Ophthalmol. 2015;15: 1–10. doi: 10.1186/s12886-015-0110-3 26383096 PMC4574621

[17] Won JY, Kim SE, Park YH. Effect of age and sex on retinal layer thickness and volume in normal eyes. Med (United States). 2016;95. doi: 10.1097/MD.0000000000005441 27861391 PMC5120948

[18] Hashmani N, Hashmani S, Murad A, Shah SMM, Hashmani M. Assessing reproducibility and the effects of demographic variables on the normal macular layers using the spectralis SD-OCT. Clin Ophthalmol. 2018;12: 1433–1440. doi: 10.2147/OPTH.S172109 30147295 PMC6095115

[19] Nieves-Moreno M, Martínez-de-la-Casa JM, Morales-Fernández L, Sánchez-Jean R, Sáenz-Francés F, García-Feijoó J. Impacts of age and sex on retinal layer thicknesses measured by spectral domain optical coherence tomography with Spectralis. PLoS One. 2018;13: e0194169. doi: 10.1371/journal.pone.0194169 29522565 PMC5844598

[20] Xu Q, Li Y, Cheng Y, Qu Y. Assessment of the effect of age on macular layer thickness in a healthy Chinese cohort using spectral-domain optical coherence tomography. BMC Ophthalmol. 2018;18: 1–9. doi: 10.1186/s12886-018-0842-y 29996804 PMC6042244

[21] Chua J, Tham YC, Tan B, Devarajan K, Schwarzhans F, Gan A, et al. Age-related changes of individual macular retinal layers among Asians. Sci Rep. 2019;9: 1–11. doi: 10.1038/s41598-019-56996-6 31889143 PMC6937292

[22] Khawaja AP, Chua S, Hysi PG, Georgoulas S, Currant H, Fitzgerald TW, et al. Comparison of Associations with Different Macular Inner Retinal Thickness Parameters in a Large Cohort: The UK Biobank. Ophthalmology. 2020;127: 62–71. doi: 10.1016/j.ophtha.2019.08.015 31585827

[23] Trinh M, Khou V, Zangerl B, Kalloniatis M, Nivison-Smith L. Modelling normal age-related changes in individual retinal layers using location-specific OCT analysis. Sci Rep. 2021;11: 1–16. doi: 10.1038/s41598-020-79424-6 33436715 PMC7804110

[24] Palazon-Cabanes A, Palazon-Cabanes B, Rubio-Velazquez E, Lopez-Bernal MD, Garcia-Medina JJ, Villegas-Perez MP. Normative Database for All Retinal Layer Thicknesses Using SD-OCT Posterior Pole Algorithm and the Effects of Age, Gender and Axial Lenght. J Clin Med. 2020;9: 3317. doi: 10.3390/jcm9103317 33076558 PMC7602827

[25] Chauhan BC, Vianna JR, Sharpe GP, Demirel S, Girkin CA, Mardin CY, et al. Differential Effects of Aging in the Macular Retinal Layers, Neuroretinal Rim, and Peripapillary Retinal Nerve Fiber Layer. Ophthalmology. 2020;127: 177–185. doi: 10.1016/j.ophtha.2019.09.013 31668716 PMC6982591

[26] Tick S, Rossant F, Ghorbel I, Gaudric A, Sahel J-A, Chaumet-Riffaud P, et al. Foveal shape and structure in a normal population. Investig Ophthalmol Vis Sci. 2011;52: 5105–5110. doi: 10.1167/iovs.10-7005 21803966

[27] Olvera-Barrios A, Kihara Y, Wu Y, Warwick N A, Müller PL, Williams KM, et al. Foveal Curvature and Its Associations in UK Biobank Participants. Invest Ophthalmol Vis Sci. 2022;63: 26. doi: 10.1167/iovs.63.8.26 35900728 PMC9344217

[28] Nesmith B, Gupta A, Strange T, Schaal Y, Schaal S. Mathematical analysis of the normal anatomy of the aging fovea. Investig Ophthalmol Vis Sci. 2014;55: 5962–5966. doi: 10.1167/iovs.14-15278 25168895

[29] Gella L, Pal SS, Ganesan S, Sharma T, Raman R. Foveal slope measurements in diabetic retinopathy: Can it predict development of sight-threatening retinopathy? Sankara Nethralaya Diabetic Retinopathy Epidemiology and Molecular Genetics Study (SN-DREAMS II, Report no 8). Indian J Ophthalmol. 2015;63: 478–481. doi: 10.4103/0301-4738.162578 26265635 PMC4550977

[30] Sepulveda JA, Turpin A, McKendrick AM. Individual differences in Foveal shape: Feasibility of individual maps between structure and function within the macular region. Investig Ophthalmol Vis Sci. 2016;57: 4772–4778. doi: 10.1167/iovs.16-19288 27623333

[31] Tewarie P, Balk L, Costello F, Green A, Martin R, Schippling S, et al. The OSCAR-IB Consensus Criteria for Retinal OCT Quality Assessment. PLoS One. 2012;7: e34823. doi: 10.1371/journal.pone.0034823 22536333 PMC3334941

[32] Ctori I, Gruppetta S, Huntjens B. The effects of ocular magnification on Spectralis spectral domain optical coherence tomography scan length. Graefe’s Arch Clin Exp Ophthalmol. 2015;253: 733–738. doi: 10.1007/s00417-014-2915-9 25572356

[33] Romero-Bascones D, Barrenechea M, Murueta-Goyena A, Galdós M, Gómez-Esteban JC, Gabilondo I, et al. Foveal Pit Morphology Characterization: A Quantitative Analysis of the Key Methodological Steps. Entropy. 2021;23: 699. doi: 10.3390/e23060699 34205877 PMC8227188

[34] Holm S. A Simple Sequentially Rejective Multiple Test Procedure. Scand J Stat. 1979;6: 65–70. Available: http://www.jstor.org/stable/4615733

[35] Benjamini Y, Hochberg Y. Controlling the False Discovery Rate: A Practical and Powerful Approach to Multiple Testing. J R Stat Soc Ser B. 1995;57: 289–300. doi: 10.1111/j.2517-6161.1995.tb02031.x

[36] Jonas JB, Schmidt AM, Muller-Bergh JA, Schlotzer-Schrehardt UM, Naumann GOH. Human optic nerve fiber count and optic disc size. Investig Ophthalmol Vis Sci. 1992;33: 2012–2018. 1582806

[37] Gao H, Hollyfield JG. Aging of the human retina: Differential loss of neurons and retinal pigment epithelial cells. Investig Ophthalmol Vis Sci. 1992;33: 1–17. 1730530

[38] Ooto S, Hangai M, Tomidokoro A, Saito H, Araie M, Otani T, et al. Effects of age, sex, and axial length on the three-dimensional profile of normal macular layer structures. Investig Ophthalmol Vis Sci. 2011;52: 8769–8779. doi: 10.1167/iovs.11-8388 21989721

[39] Mauschitz MM, Holz FG, Finger RP, Breteler MMB. Determinants of Macular Layers and Optic Disc Characteristics on SD-OCT: The Rhineland Study. Transl Vis Sci Technol. 2019;8: 34. doi: 10.1167/tvst.8.3.34 31183250 PMC6549562

[40] Demirkaya N, van Dijk HW, van Schuppen SM, Abràmoff MD, Garvin MK, Sonka M, et al. Effect of age on individual retinal layer thickness in normal eyes as measured with spectral-domain optical coherence tomography. Investig Ophthalmol Vis Sci. 2013;54: 4934–4940. doi: 10.1167/iovs.13-11913 23761080 PMC5963176

[41] Yoshioka N, Zangerl B, Nivison-Smith L, Khuu SK, Jones BW, Pfeiffer RL, et al. Pattern recognition analysis of age-related retinal ganglion cell signatures in the human eye. Investig Ophthalmol Vis Sci. 2017;58: 3086–3099. doi: 10.1167/iovs.17-21450 28632847 PMC5482244

[42] Tong J, Phu J, Khuu SK, Yoshioka N, Choi AY, Nivison-Smith L, et al. Development of a Spatial Model of Age-Related Change in the Macular Ganglion Cell Layer to Predict Function From Structural Changes. Am J Ophthalmol. 2019;208: 166–177. doi: 10.1016/j.ajo.2019.04.020 31078539 PMC6842123

[43] Oberwahrenbrock T, Weinhold M, Mikolajczak J, Zimmermann H, Paul F, Beckers I, et al. Reliability of intra-retinal layer thickness estimates. PLoS One. 2015;10: 1–16. doi: 10.1371/journal.pone.0137316 26349053 PMC4562656

[44] Menke MN, Dabov S, Knecht P, Sturm V. Reproducibility of Retinal Thickness Measurements in Healthy Subjects Using Spectralis Optical Coherence Tomography. Am J Ophthalmol. 2009;147: 467–472. doi: 10.1016/j.ajo.2008.09.005 19026403

[45] Barrio-Barrio J, Noval S, Galdós M, Ruiz-Canela M, Bonet E, Capote M, et al. Multicenter Spanish study of spectral-domain optical coherence tomography in normal children. Acta Ophthalmol. 2013;91: 56–63. doi: 10.1111/j.1755-3768.2012.02562.x 23347665

[46] Pope JM, Verkicharla PK, Sepehrband F, Suheimat M, Schmid KL, Atchison DA. Three-dimensional MRI study of the relationship between eye dimensions, retinal shape and myopia. Biomed Opt Express. 2017;8: 2386. doi: 10.1364/BOE.8.002386 28663880 PMC5480487

[47] Szigeti A, Tátrai E, Varga BE, Szamosi A, DeBuc DC, Nagy ZZ, et al. The Effect of Axial Length on the Thickness of Intraretinal Layers of the Macula. PLoS One. 2015;10: e0142383. doi: 10.1371/journal.pone.0142383 26544553 PMC4636257

[48] Kuo AN, McNabb RP, Chiu SJ, El-Dairi MA, Farsiu S, Toth CA, et al. Correction of ocular shape in retinal optical coherence tomography and effect on current clinical measures. Am J Ophthalmol. 2013;156: 304–311. doi: 10.1016/j.ajo.2013.03.012 23659972 PMC3854927

[49] Straub J, Steidle M. Estimating the shape of the human eye using widefield optical coherence tomography (OCT). Proc SPIE 10685, Biophotonics: Photonic Solutions for Better Health Care VI, 106851V. 2018. doi: 10.1117/12.2306604

[50] Hoffer KJ, Savini G. Effect of Gender and Race on Ocular Biometry. Int Ophthalmol Clin. 2017;57: 137–142. doi: 10.1097/IIO.0000000000000180 28590287

[51] Dubis AM, Hansen BR, Cooper RF, Beringer J, Dubra A, Carroll J. Relationship between the foveal avascular zone and foveal pit morphology. Investig Ophthalmol Vis Sci. 2012;53: 1628–1636. doi: 10.1167/iovs.11-8488 22323466 PMC3339921

[52] Breher K, Agarwala R, Leube A, Wahl S. Direct modeling of foveal pit morphology from distortion-corrected OCT images. Biomed Opt Express. 2019;10: 4815–4824. doi: 10.1364/BOE.10.004815 31565527 PMC6757460

[53] Denniss J, Turpin A, McKendrick AM. Individualized structure-function mapping for glaucoma: Practical constraints on map resolution for clinical and research applications. Investig Ophthalmol Vis Sci. 2014;55: 1985–1993. doi: 10.1167/iovs.13-13758 24557345

