# Supplementary figures and images for: Spatial characterization of the effect of age and sex on macular layer thicknesses and foveal pit morphology
Source: PLoS One. 2022 Dec 15;17(12):e0278925. doi: 10.1371/journal.pone.0278925 (PMC9754220; doi:10.1371/journal.pone.0278925)

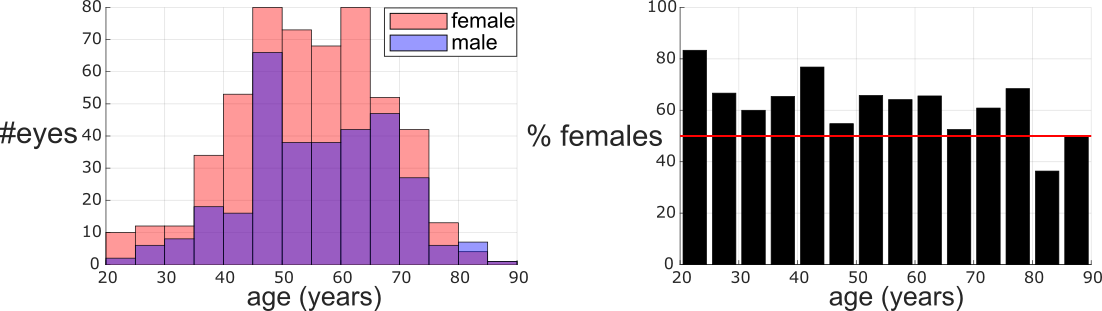

Supplement: S1 Fig — (TIF) [file pone.0278925.s001.tif]

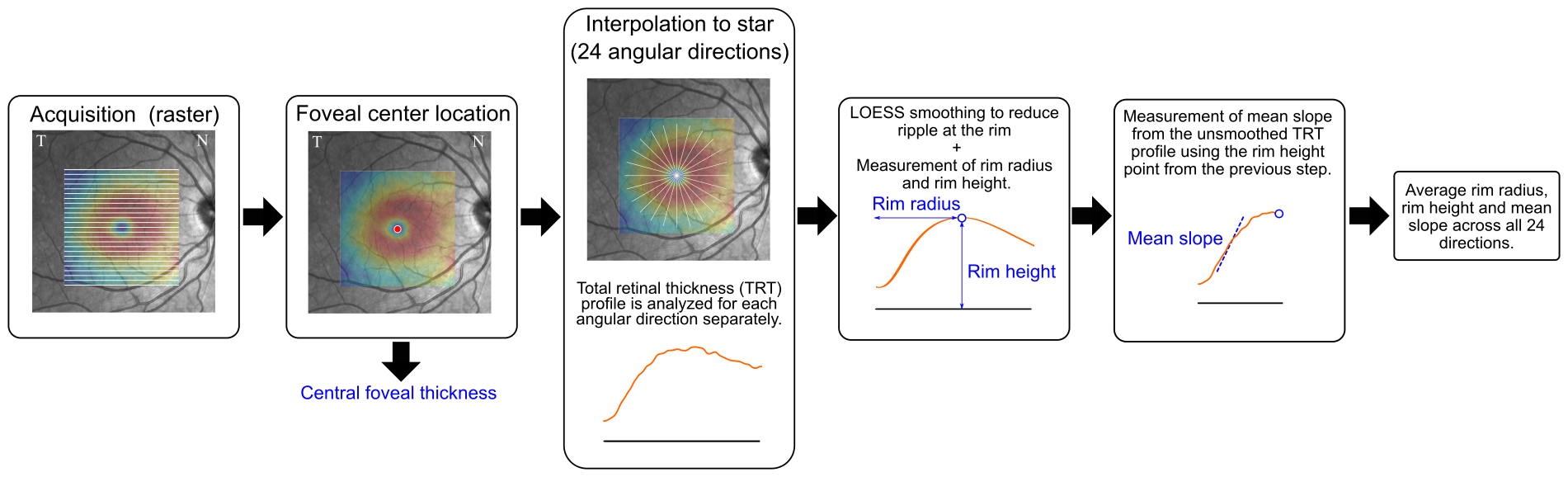

Supplement: S2 Fig — (TIF) [file pone.0278925.s002.tif]

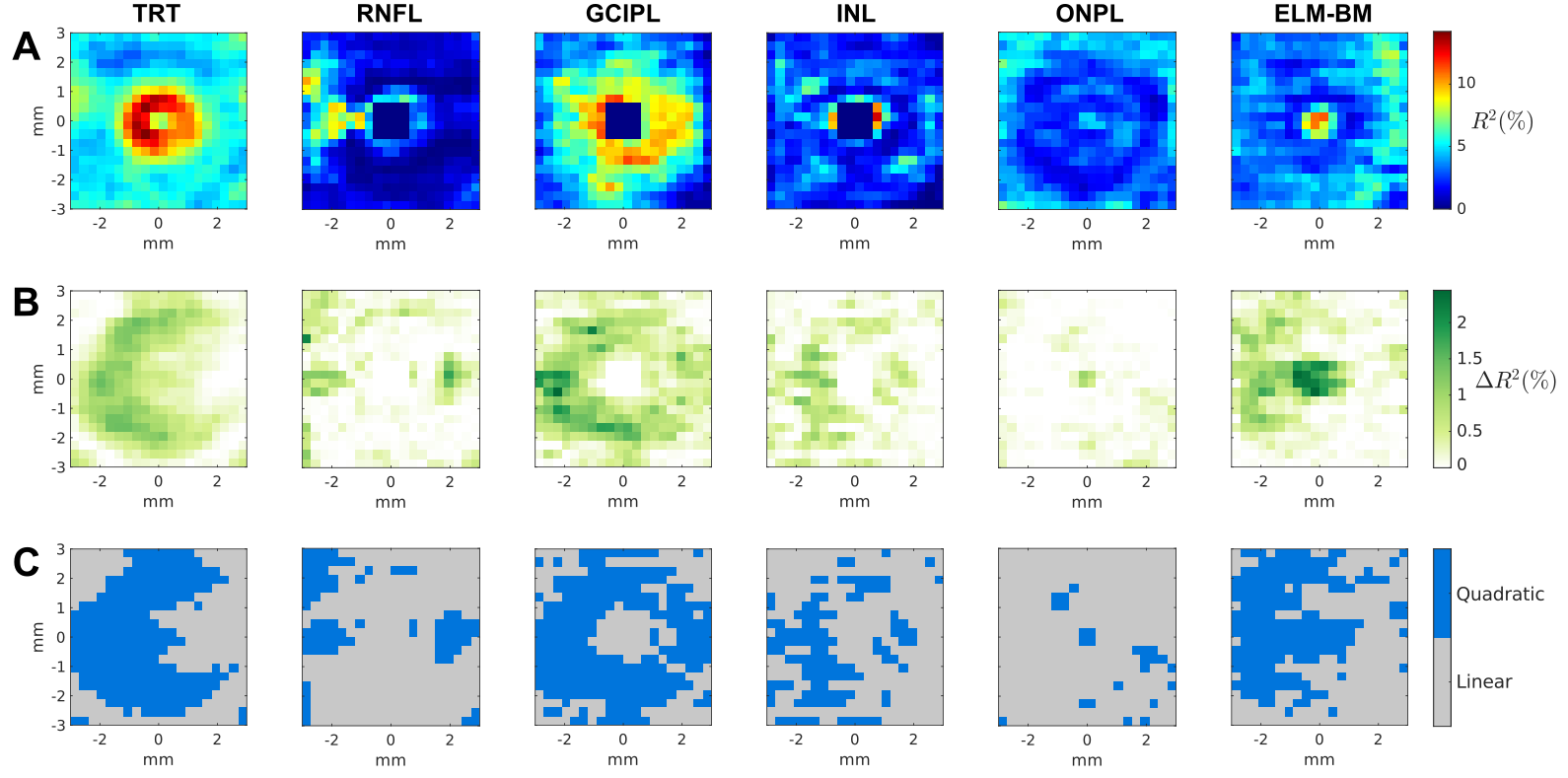

Supplement: S3 Fig — (A) R-squared of the linear model (Eq 1 in the paper). (B) Improvement on the R-squared when a quadratic term for age is added (βage2·age2). (C) Model selected for each sector. Layers under study: total retinal thickness (TRT), retinal nerve fiber layer (RNFL), ganglion cell–inner plexiform layer (GCIPL), inner nuclear layer (INL), outer nuclear and plexiform (ONPL), and external limiting membrane–Bruch’s membrane complex (ELM-BM). (TIF) [file pone.0278925.s003.tif]

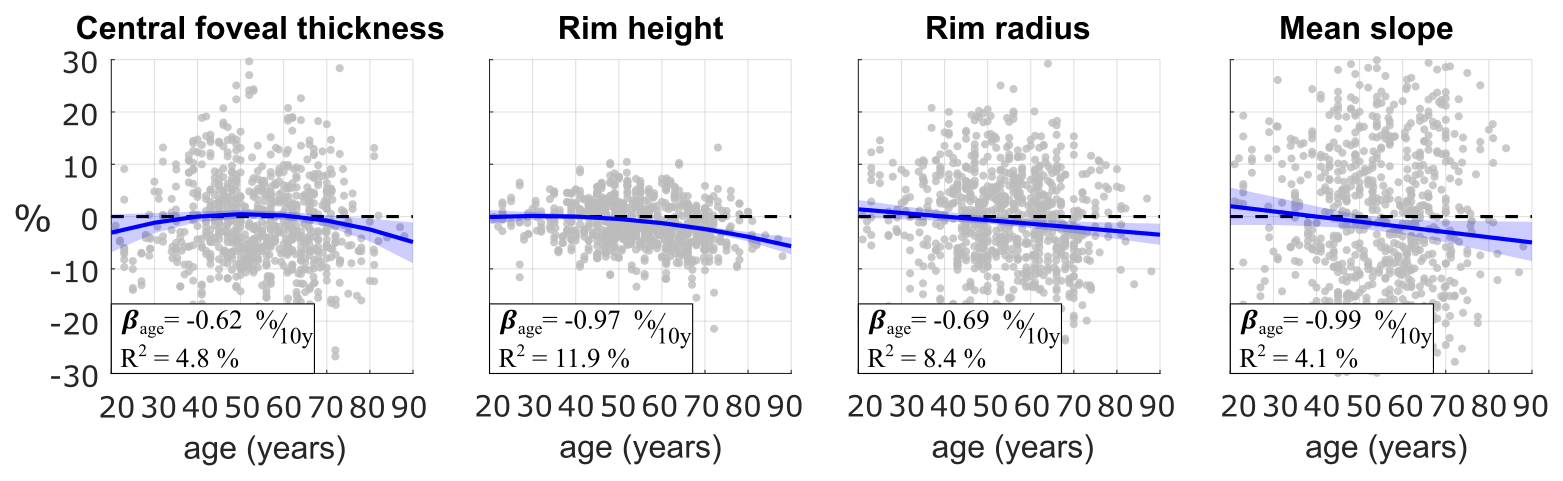

Supplement: S4 Fig — Each parameter value was obtained after averaging 24 angular directions and transformed into percentages as the relative difference with respect to the average value of the youngest group (age < 40). (TIF) [file pone.0278925.s004.tif]

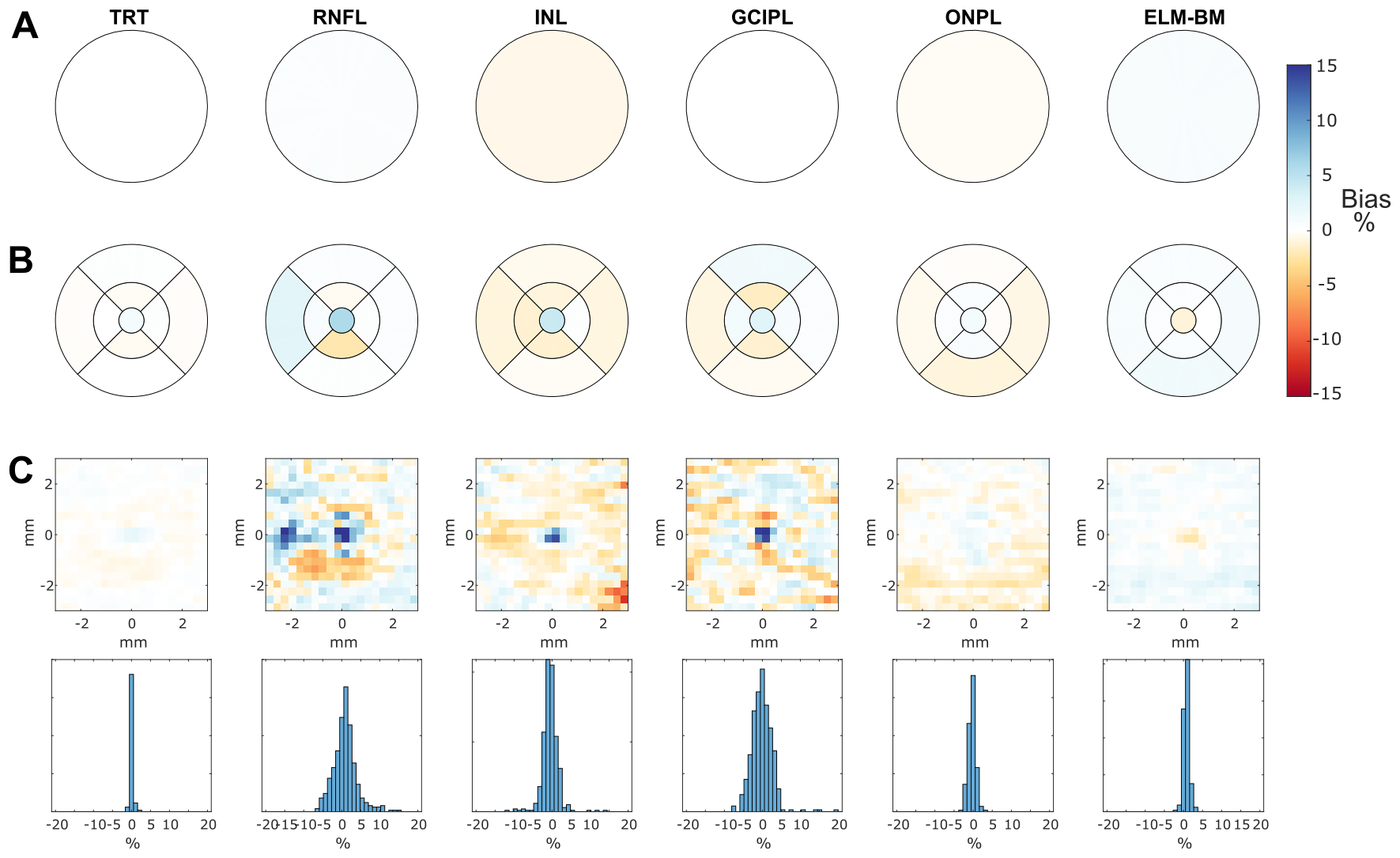

Supplement: S5 Fig — Percentual bias between regular and high-resolution acquisition protocols for the whole macula (A), the ETDRS sectors (B) and the 20 x 20 grid (C). The latter includes the distribution of individual sector values (bottom row). (TIF) [file pone.0278925.s005.tif]
