# Supplementary material for: Spatial characterization of the effect of age and sex on macular layer thicknesses and foveal pit morphology
Source: PLoS One. 2022 Dec 15;17(12):e0278925. doi: 10.1371/journal.pone.0278925 (PMC9754220; doi:10.1371/journal.pone.0278925)
Supplement: S1 Appendix — (DOCX) [file pone.0278925.s006.docx]

**S1 Appendix. Results for ETDRS sectorization.**

**Table 1. Absolute age effect (µm/ 10 years) on ETDRS sectors.**

| **Layer** | **C** | **IN** | **IS** | **IT** | **II** | **ON** | **OS** | **OT** | **OI** |
| --- | --- | --- | --- | --- | --- | --- | --- | --- | --- |
| TRT | -1.88** | -2.54** | -3.49** | -3.31** | -3.67** | -3.06** | -3.3** | -2.87** | -3.61** |
| RNFL | 0.05 | 0.14** | 0.07 | 0.28** | -0.04 | 0.09 | 0.42** | 0.68** | 0.01 |
| GCIP | -0.38** | -2.33** | -2.14** | -2.04** | -2.32** | -1.85** | -1.6** | -1.77** | -1.7** |
| INL | 0.59** | 0.18** | 0.03 | -0.31** | -0.07 | -0.56** | -0.46** | -0.73** | -0.46** |
| ONPL | -0.33** | -0.59** | -0.93** | -0.56** | -0.54** | -0.74** | -0.98** | -0.58** | -0.64** |
| ELM-BM | -1.66** | -0.6** | -0.22** | -0.59** | -0.33** | -0.35** | -0.35** | -0.4** | -0.5** |

* p<0.05, ****** p < 0.05 after FDR correction**.** Abbreviations: TRT: total retinal thickness; RNFL: retinal nerve fiber layer; GCIPL: ganglion cell‒inner plexiform layer; INL: inner nuclear layer; ONPL: outer nuclear and plexiform layer; ELM-BM: external limiting membrane‒Bruch’s membrane layer complex; C: central; IN: inner nasal; IS: inner superior; IT: inner temporal; II: inner inferior; ON: outer nasal; OS: outer superior; OT outer temporal; OI: outer inferior.

**Table 2. Normalized age effect (% / 10 years) on ETDRS sectors.**

| **Layer** | **C** | **IN** | **IS** | **IT** | **II** | **ON** | **OS** | **OT** | **OI** |
| --- | --- | --- | --- | --- | --- | --- | --- | --- | --- |
| TRT | -0.68** | -0.73** | -1** | -0.99** | -1.06** | -0.95** | -1.08** | -1** | -1.23** |
| RNFL | 0.45 | 0.67** | 0.29 | 1.65** | -0.15 | 0.2 | 1.12** | 3.75** | 0.02 |
| GCIP | -1.09** | -2.37** | -2.18** | -2.18** | -2.38** | -2.61** | -2.4** | -2.54** | -2.69** |
| INL | 3.29** | 0.45** | 0.08 | -0.82** | -0.18 | -1.59** | -1.44** | -2.14** | -1.47** |
| ONPL | -0.27** | -0.54** | -0.89** | -0.53** | -0.53** | -0.85** | -1.11** | -0.68** | -0.8** |
| ELM-BM | -1.8** | -0.71** | -0.27** | -0.72** | -0.4** | -0.44** | -0.43** | -0.51** | -0.64** |

* p<0.05, ****** p < 0.05 after FDR correction**.** Abbreviations: TRT, total retinal thickness; RNFL: retinal nerve fiber layer; GCIPL: ganglion cell‒inner plexiform layer; INL: inner nuclear layer; ONPL: outer nuclear and plexiform layer; ELM-BM: external limiting membrane‒Bruch’s membrane layer complex; C: central; IN: inner nasal; IS: inner superior; IT: inner temporal; II: inner inferior; ON: outer nasal; OS: outer superior; OT outer temporal; OI: outer inferior.

**Table 3. Absolute sex effect (µm difference towards males) on ETDRS sectors.**

| **Layer** | **C** | **IN** | **IS** | **IT** | **II** | **ON** | **OS** | **OT** | **OI** |
| --- | --- | --- | --- | --- | --- | --- | --- | --- | --- |
| TRT | 13.9** | 9.48** | 7.59** | 9.26** | 7.88** | 2.46** | 1.6** | 4.61** | 0.98 |
| RNFL | 1.72** | 0.94** | 0.8** | 0.37** | 0.87** | -0.04 | -0.75** | 0.2** | -0.79** |
| GCIP | 4.14** | 2.6** | 2.03** | 3.51** | 1.76** | -0.53 | -0.3 | 0.91** | -0.68** |
| INL | 4.41** | 2.61** | 2.09** | 2.39** | 1.87** | 0.04 | 0.45** | 0.54** | 0.19 |
| ONPL | 4.21** | 2.86** | 1.81** | 2.33** | 2.51** | 1.77** | 1.22** | 1.87** | 1.28** |
| ELM-BM | -0.55** | 0.53** | 0.81** | 0.61** | 0.8** | 1.26** | 0.95** | 1.08** | 0.99** |

* p<0.05, ** p < 0.05 after false discovery rate correction. Abbreviations: TRT: total retinal thickness; RNFL: retinal nerve fiber layer; GCIPL: ganglion cell‒inner plexiform layer; INL: inner nuclear layer; ONPL: outer nuclear and plexiform layer; ELM-BM: external limiting membrane‒Bruch’s membrane complex; C: central; IN: inner nasal; IS: inner superior; IT: inner temporal; II: inner inferior; ON: outer nasal; OS: outer superior; OT outer temporal; OI: outer inferior.

**Table 4. Normalized sex effect (% difference towards males) on ETDRS sectors.**

| **Layer** | **C** | **IN** | **IS** | **IT** | **II** | **ON** | **OS** | **OT** | **OI** |
| --- | --- | --- | --- | --- | --- | --- | --- | --- | --- |
| TRT | 5.15** | 2.74** | 2.19** | 2.8** | 2.3** | 0.77** | 0.53** | 1.62** | 0.34 |
| RNFL | 16.12** | 4.68** | 3.37** | 2.25** | 3.44** | -0.08 | -2** | 1.1** | -1.98** |
| GCIP | 12.54** | 2.68** | 2.08** | 3.81** | 1.82** | -0.74 | -0.45 | 1.32** | -1.07** |
| INL | 28.27** | 6.77** | 5.32** | 6.56** | 4.71** | 0.11 | 1.41** | 1.59** | 0.61 |
| ONPL | 3.56** | 2.68** | 1.74** | 2.25** | 2.51** | 2.05** | 1.39** | 2.22** | 1.61** |
| ELM-BM | -0.59** | 0.63** | 0.99** | 0.74** | 0.98** | 1.6** | 1.19** | 1.37** | 1.28** |

* p<0.05, ** p < 0.05 after false discovery rate correction. Abbreviations: TRT, total retinal thickness; RNFL: retinal nerve fiber layer; GCIPL: ganglion cell‒inner plexiform layer; INL: inner nuclear layer; ONPL: outer nuclear and plexiform layer; ELM-BM: external limiting membrane‒Bruch’s membrane complex; C: central; IN: inner nasal; IS: inner superior; IT: inner temporal; II: inner inferior; ON: outer nasal; OS: outer superior; OT outer temporal; OI: outer inferior.

**Table 5: Full regression results for mean macula and pit parameters**

| Category | Parameter | Age  dependence | β_0_ | β_age_ | β_age2_ | β_sex_ (male) | β_scan_focus_ | R^2^ |
| --- | --- | --- | --- | --- | --- | --- | --- | --- |
| Thickness  (µm) | TRT | Quadratic | 302.68  [287, 318.35] | 0.39  [-0.2, 0.98] | -0.006  [-0.011, -0.001] | 4.14  [-4.09, 6.5] | -0.155  [1.782, 0.222] | 8.2 |
|  | RNFL | Linear | 31.08  [29.59, 32.56] | 0.02  [0, 0.05] | - | -0.03  [-0.04, 0.65] | 0.005  [-0.711, 0.151] | 0.2 |
|  | GCIP | Quadratic | 69.98  [63.43, 76.53] | 0.21  [-0.04, 0.45] | -0.003  [-0.005, -0.001] | 0.57  [-2.13, 1.56] | -0.331  [-0.412, -0.176] | 11.9 |
|  | INL | Quadratic | 33.01  [30.3, 35.72] | 0.05  [-0.05, 0.16] | -0.001  [-0.002, 0] | 0.87  [-0.6, 1.28] | 0.004  [0.461, 0.091] | 7.1 |
|  | ONPL | Linear | 92.46  [89.83, 95.09] | -0.07  [-0.11, -0.02] | - | 1.8  [-1.12, 3.02] | -0.109  [0.592, 0.072] | 2.6 |
|  | ELM_BM | Quadratic | 78.32  [75.27, 81.37] | 0.08  [-0.03, 0.2] | -0.001  [-0.002, 0] | 0.93 [-0.63, 1.39] | 0.171  [0.477, 0.269] | 6.0 |
| Foveal pit morphology | CFT (µm) | Quadratic | 209.23  [184.1, 234.35] | 0.86  [-0.08, 1.81] | -0.01  [-0.02, 0] | 7.99  [4.22, 11.77] | -0.98  [-1.77, -0.18] | 4.8 |
|  | Rim height (µm) | Quadratic | 345.1  [327.2, 363.01] | 0.37  [-0.3, 1.05] | -0.01  [-0.01, 0] | 8.63  [5.94, 11.33] | -0.11  [-0.57, 0.35] | 11.9 |
|  | Rim radius (µm) | Linear | 1165.05  [1123.81, 1206.28] | -0.77  [-1.5, -0.03] | - | -59.4  [-78.34, -40.46] | -4.9  [-8.62, -1.19] | 8.4 |
|  | Mean slope (º) | Linear | 6.5  [6.03, 6.96] | -0.01  [-0.01, 0] | - | 0.39  [0.18, 0.6] | 0.07  [0.03, 0.12] | 4.1 |

* Values with 95% confidence interval. Abbreviations: TRT, total retinal thickness; RNFL: retinal nerve fiber layer; GCIPL: ganglion cell‒inner plexiform layer; INL: inner nuclear layer; ONPL: outer nuclear and plexiform layer; ELM-BM: external limiting membrane‒Bruch’s membrane complex; C: central; IN: inner nasal; IS: inner superior; IT: inner temporal; II: inner inferior; ON: outer nasal; OS: outer superior; OT outer temporal; OI: outer inferior.

**Table 6. Absolute bias between standard (25 B-scan, 496 A-scan) and high-resolution (97 B-scan, 1024 A-scan) protocols**

| **Layer** | **C** | **IN** | **IS** | **IT** | **IF** | **ON** | **OS** | **OT** | **OI** |
| --- | --- | --- | --- | --- | --- | --- | --- | --- | --- |
| TRT | 2.89  [1.35, 4.42] | 0.42  [-1.44, 2.29] | -0.62  [-2.74, 1.5] | 0.11  [-1.44, 1.67] | -1.17  [-3.02, 0.68] | -0.09  [-1.26, 1.07] | 0.65  [-1.25, 2.55] | -0.17  [-1.21, 0.87] | 0.11  [-1.12, 1.33] |
| RNFL | 0.64  [0.24, 1.04] | 0.02  [-0.59, 0.64] | -0.07  [-0.72, 0.59] | 0.1  [-0.27, 0.47] | -0.56  [-1.22, 0.1] | 0.19  [-0.85, 1.22] | 0.14  [-0.79, 1.06] | 0.45  [0.03, 0.87] | 0.08  [-0.74, 0.89] |
| GCIP | 1.39  [0.71, 2.07] | 0.16  [-1.12, 1.45] | -0.84  [-2.4, 0.73] | -1.1  [-2.14, -0.07] | -1.19  [-2.1, -0.29] | -0.55  [-1.38, 0.28] | -0.29  [-1.06, 0.48] | -0.6  [-1.26, 0.06] | -0.11  [-0.78, 0.55] |
| INL | 0.41  [-0.16, 0.99] | 0.29  [-0.5, 1.09] | -0.61  [-1.78, 0.55] | 0.38  [-0.29, 1.04] | -0.5  [-1.68, 0.68] | 0.14  [-0.25, 0.52] | 0.39  [-0.19, 0.96] | -0.26  [-0.67, 0.16] | -0.09  [-0.5, 0.31] |
| ONPL | 1.35  [-0.09, 2.8] | -0.09  [-1.01, 0.82] | 0.59  [-0.47, 1.65] | 0.39  [-0.31, 1.1] | 0.69  [-0.5, 1.89] | -0.59  [-1.14, -0.04] | -0.01  [-0.84, 0.81] | -0.35  [-0.91, 0.21] | -0.73  [-1.32, -0.15] |
| ELM_BM | -0.9  [-2.15, 0.35] | 0.03  [-0.84, 0.91] | 0.31  [-0.38, 1] | 0.35  [-0.24, 0.94] | 0.39  [-0.34, 1.13] | 0.73  [0.09, 1.36] | 0.42  [-0.2, 1.05] | 0.58  [0.02, 1.14] | 0.97  [0.47, 1.48] |

* Absolute difference with 95% confidence intervals. Abbreviations: TRT, total retinal thickness; RNFL: retinal nerve fiber layer; GCIPL: ganglion cell‒inner plexiform layer; INL: inner nuclear layer; ONPL: outer nuclear and plexiform layer; ELM-BM: external limiting membrane‒Bruch’s membrane complex; C: central; IN: inner nasal; IS: inner superior; IT: inner temporal; II: inner inferior; ON: outer nasal; OS: outer superior; OT outer temporal; OI: outer inferior.

**Table 7. Relative bias between standard (25 B-scan, 496 A-scan) and high-resolution (97 B-scan, 1024 A-scan) protocols**

| **Layer** | **C** | **IN** | **IS** | **IT** | **IF** | **ON** | **OS** | **OT** | **OI** |
| --- | --- | --- | --- | --- | --- | --- | --- | --- | --- |
| TRT | 1.07  [0.5, 1.64] | 0.12  [-0.42, 0.66] | -0.18  [-0.8, 0.44] | 0.03  [-0.44, 0.51] | -0.34  [-0.89, 0.2] | -0.03  [-0.39, 0.34] | 0.22  [-0.42, 0.85] | -0.06  [-0.43, 0.31] | 0.04  [-0.39, 0.46] |
| RNFL | 5.82  [2.17, 9.47] | 0.12  [-2.91, 3.15] | -0.28  [-3.07, 2.51] | 0.59  [-1.62, 2.81] | -2.19  [-4.77, 0.38] | 0.39  [-1.79, 2.58] | 0.38  [-2.24, 3.01] | 2.57  [0.16, 4.97] | 0.2  [-1.87, 2.26] |
| GCIP | 4.16  [2.12, 6.21] | 0.18  [-1.2, 1.55] | -0.89  [-2.55, 0.78] | -1.23  [-2.38, -0.08] | -1.27  [-2.24, -0.31] | -0.81  [-2.03, 0.41] | -0.46  [-1.67, 0.76] | -0.9  [-1.9, 0.09] | -0.19  [-1.32, 0.93] |
| INL | 2.66  [-1.06, 6.38] | 0.77  [-1.34, 2.89] | -1.56  [-4.51, 1.39] | 1.07  [-0.81, 2.95] | -1.27  [-4.25, 1.72] | 0.4  [-0.71, 1.51] | 1.25  [-0.62, 3.13] | -0.78  [-2.05, 0.49] | -0.3  [-1.64, 1.03] |
| ONPL | 1.15  [-0.08, 2.37] | -0.08  [-0.91, 0.75] | 0.56  [-0.44, 1.56] | 0.37  [-0.3, 1.04] | 0.68  [-0.49, 1.85] | -0.66  [-1.28, -0.04] | -0.01  [-0.92, 0.9] | -0.4  [-1.04, 0.24] | -0.9  [-1.62, -0.19] |
| ELM_BM | -0.98  [-2.35, 0.38] | 0.04  [-1.01, 1.09] | 0.38  [-0.47, 1.22] | 0.43  [-0.3, 1.15] | 0.49  [-0.43, 1.41] | 0.92  [0.11, 1.73] | 0.54  [-0.25, 1.32] | 0.76  [0.03, 1.49] | 1.27  [0.61, 1.93] |

* Values in % with 95% confidence interval. Abbreviations: TRT, total retinal thickness; RNFL: retinal nerve fiber layer; GCIPL: ganglion cell‒inner plexiform layer; INL: inner nuclear layer; ONPL: outer nuclear and plexiform layer; ELM-BM: external limiting membrane‒Bruch’s membrane complex; C: central; IN: inner nasal; IS: inner superior; IT: inner temporal; II: inner inferior; ON: outer nasal; OS: outer superior; OT outer temporal; OI: outer inferior.
