# Supplementary material for: Spatial characterization of the effect of age and sex on macular layer thicknesses and foveal pit morphology
Source: PLoS One. 2022 Dec 15;17(12):e0278925. doi: 10.1371/journal.pone.0278925 (PMC9754220; doi:10.1371/journal.pone.0278925)
Supplement: S1 Table — *p<0.05. Abbreviations: CFT: central foveal thickness; NAF: no association found, estimations not reported. †In the present study the mean slope was studied instead of the maximum slope. Olvera-Barrios et al. measured foveal curvature instead of slope. (DOCX) [file pone.0278925.s007.docx]

| **Study** | | | **N** | | **Age** | | **ΔCFT**  (µm / 10 years) | **ΔRim-height** (µm / 10 years) | **ΔDepth**  (µm / 10 years) | **ΔRim-radius**  (µm / 10 years) | **ΔSlope^†^**  (º / 10 years) |
| --- | --- | --- | --- | --- | --- | --- | --- | --- | --- | --- | --- |
|  |  |  |  |  | Mean ± sd | Range |  |  |  |  |  |
| Present study | | | 444 | | 54.9 ± 12.7 | 21-88 | -1.44 | -3.42* | - | -7.66* | -0.06 |
| Tick, 2011^26^ | | | 57 | | - | 18-45 | NAF | NAF | NAF | NAF | - |
| Nesmith, 2014^28^ | | | 390 | | 52.6 | 13-97 | - | - | - | - | Increase* |
| Gella, 2015^29^ | | | 668 | | - | ≥ 40 | - | - | - | - | Decrease* |
| Sepulveda, 2016^30^ | Young | 20 | | - | | 24-33 | NAF | NAF | - | Decrease | - |
|  | Old | 10 | | - | | 62-76 |  |  |  |  |  |
| Zouache, 2020^12^ | Ghanaian | 84 | | 65.1 ± 9.4 | | 45-82 | - | - | -4.8 | -46* | -0.054* |
|  | Caucasian | 37 | | 61.9 ± 11.5 | | 41-85 | - | - | -1.5 | +30 | -0.012 |
| Olvera-Barrios, 2022^27^ | | 63939 | | 56 ± 8.0 | | 40-69 | - | - | - | - | Increase in females* |
