# Supplementary material for: Spatial characterization of the effect of age and sex on macular layer thicknesses and foveal pit morphology
Source: PLoS One. 2022 Dec 15;17(12):e0278925. doi: 10.1371/journal.pone.0278925 (PMC9754220; doi:10.1371/journal.pone.0278925)
Supplement: S2 Table — The results are reported as the difference in the mean values of males minus females. *p<0.05. Abbreviations: CFT: central foveal thickness. † In the present study the mean slope was studied instead of the maximum slope. Olvera-Barrios et al. measured foveal curvature instead of slope. (DOCX) [file pone.0278925.s008.docx]

| **Study** | | **N ♂** | **N ♀** | **Age** | | **CFT**  **(µm)** | **Rim height (µm)** | **Pit depth (µm)** | **Rim radius**  **(µm)** | **Slope^†^**  **(º)** |
| --- | --- | --- | --- | --- | --- | --- | --- | --- | --- | --- |
|  |  |  |  | **Mean ± sd** | **Range** |  |  |  |  |  |
| Present study | | 163 | 281 | 54.9 ± 12.7 | 21-88 | +7.99 | +8.63* | - | -59.4* | +0.39* |
| Wagner-Schuman, 2011^10^ | | 47 | 43 | 27.8 ± 9.0 | ≥ 18 | - | - | +1 | -15 | + 0.4 |
| Dubis, 2012^51^ | | 26 | 16 | 26.5 | 18-67 | - | - | +13 | +79 | +0.5 |
| Scheibe, 2016^11^ | | 109 | 111 | 44 ± 13  44±14 | 21-77 | +4.2 | +6.0* | - | -27.5 | + 0.5 |
| Zouache, 2020^12^ | Ghanaian | 30 | 54 | 65.1 ± 9.4 | 45-82 | - | - | +7 | -145 | +2.26 |
|  | Caucasian | 9 | 28 | 61.9 ± 11.5 | 41-85 | - | - | -10 | -180 | +1.05 |
| Olvera-Barrios, 2022^27^ | | 63939 | 63939 | 56 ± 8 | 40-69 | +6.8* | - | - | - | +0.87* |
